# The Timing Sequence and Mechanism of Aging in Endocrine Organs

**DOI:** 10.3390/cells12070982

**Published:** 2023-03-23

**Authors:** He Yang, Bing Fang, Zixu Wang, Yaoxing Chen, Yulan Dong

**Affiliations:** 1 Key Laboratory of Precision Nutrition and Food Quality, Department of Nutrition and Health, Ministry of Education, China Agricultural University, Beijing 100193, China; 2 Beijing Advanced Innovation Center for Food Nutrition and Human Health, Department of Nutrition and Health, China Agricultural University, Beijing 100193, China; 3 College of Veterinary Medicine, China Agricultural University, Beijing 100193, China

**Keywords:** endocrine, aging, mechanisms

## Abstract

The world is increasingly aging, and there is an urgent need to find a safe and effective way to delay the aging of the body. It is well known that the endocrine glands are one of the most important organs in the context of aging. Failure of the endocrine glands lead to an abnormal hormonal environment, which in turn leads to many age-related diseases. The aging of endocrine glands is closely linked to oxidative stress, cellular autophagy, genetic damage, and hormone secretion. The first endocrine organ to undergo aging is the pineal gland, at around 6 years old. This is followed in order by the hypothalamus, pituitary gland, adrenal glands, gonads, pancreatic islets, and thyroid gland. This paper summarises the endocrine gland aging-related genes and pathways by bioinformatics analysis. In addition, it systematically summarises the changes in the structure and function of aging endocrine glands as well as the mechanisms of aging. This study will advance research in the field of aging and help in the intervention of age-related diseases.

## 1. Introduction

**Figure 1:**
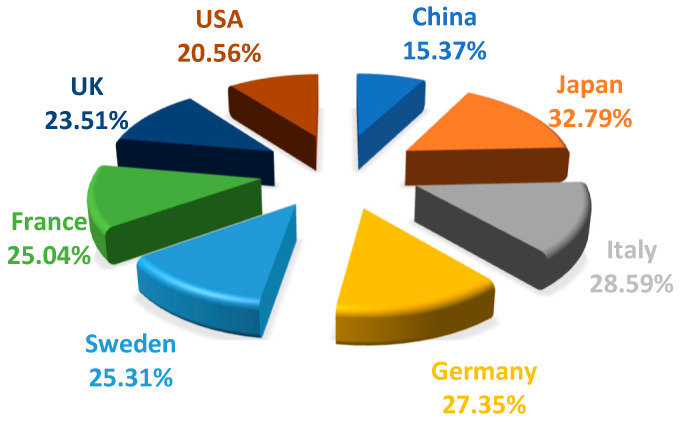
Distribution of the proportion of the population over 60.

People over the age of 60 are generally defined as elderly. Since the turn of the twenty-first century, the rate of global “aging” has increased. A total of 92 countries have become aging societies. Japan is the nation that is aging the most quickly, with more than one-third of the population over 60. The order of the proportion of the world’s population that possess aging societies is Japan, Italy, Germany, Sweden, France, UK, USA, and China [1] (Figure 1). China has been an aging society since 1999. According to the seventh national census in 2020, China’s population is as high as 1.41 billion, and people over 60 number at 217 million. Moreover, around 1.88 billion people on earth are suffering from chronic diseases [2]. The “Healthy China 2030” Planning Outline issued by the State Council advised that the total scale of the health service industry will reach RMB 16 trillion by 2030 [3]. As such, the National Health Commission issued the Healthy China Action (2019–2030). Furthermore, China is committed to the growth of a significant health care sector.

To our best knowledge, there is no systematic review of the timing sequence of endocrine glandular aging, nor the mechanisms that affect aging. In order to have a systematic understanding of endocrine glandular aging mechanisms and to promote in-depth research on age-related diseases, the study described in this paper uses bioinformatics methods to find essential genes, protein interactions, and key regulatory pathways for endocrine organ and tissue aging, which provide essential ideas for subsequent in-depth research on anti-aging. In addition, this paper focuses on the timing sequency and mechanisms of aging.

## 2. Signaling Pathways and Related Protein Interactions in Endocrine Glands Aging by Bioinformatics

**Figure 2:**
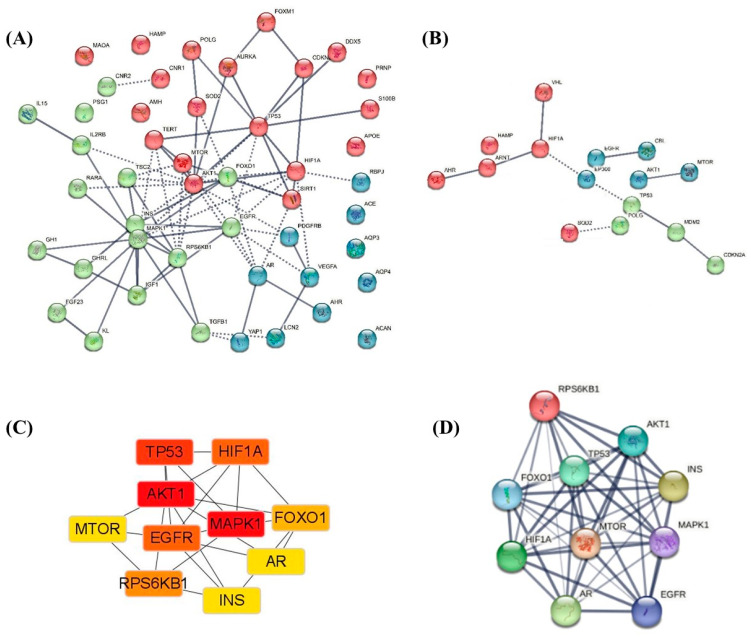
The key genes of endocrine glands aging and the protein-protein interaction (PPI) network. (**A**) The PPI network of the aging endocrine glands genes. Using the STRING online database, 44 genes were selected and used to construct the PPI network. (**B**) The PPI network of the significant genes by experiment. (**C**) The top 10 significant genes. (**D**) The PPI network of the top 10 genes.

**Figure 3:**
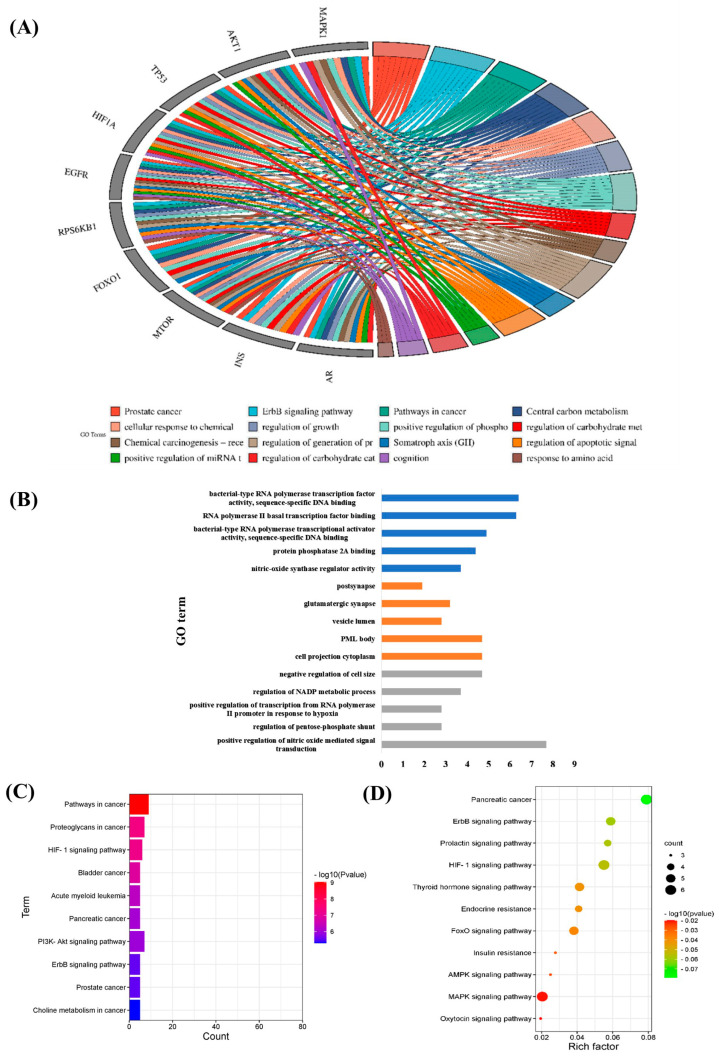
The Gene Ontology (GO) and the Kyoto Encyclopedia of Genes and Genomes (KEGG) pathway enrichment analyses of 10 significant genes. (**A**) The GO enrichment analyses of 10 genes. (**B**) The GO enrichment analyses of the top five key pathways. The GO terms were classified into three categories: biological process (BP), cellular component (CC), and molecular function (MF). These correspond to the blue, orange, and gray columns, respectively. (**C**) The top 10 pathways were enriched in KEGG. (**D**) The enrichment analysis was conducted on the KEGG pathways that relate to endocrine aging.

In this study, a search for “endocrine aging” in NCBI’s Gene was conducted and 44 genes that are related to humans were obtained. Through the String database, the protein-protein interaction (PPI) network diagram relationships were obtained. In addition, the PPI network diagram proved by the current experiments and the PPI network diagram obtained by data inference were obtained through conditional screening (Figure 2A,B). The top 10 genes were obtained from 44 genes using Cytihubba’s analysis algorithm. Again through the String database, the PPI network map was then obtained (Figure 2C,D). The GO terms were obtained using the Metascape database (Figure 3A), and the Gene Ontology and the Kyoto Encyclopedia of Genes and Genomes (KEGG) pathways were obtained via the David database (Figure 3B–D).

The top ten key genes are namely *AKT1, MAPK1, TP53, EGFR, HIF1A, RPS6KB1, FOXO1, MTOR, INS and INS.* There are five significant pathways in Biological Process (BP), Cellular Component (CC) and Molecular Function (MF). Based on significant pathways, a GO enrichment pathway map was drawn in Figure 3A. As shown in Figure 3B, the key pathway in BP is positive regulation of nitric oxide mediated signal transduction. The key pathway in CC is cell projection cytoplasm. The key pathway in MF is bacterial-type RNA polymerase transcription factor activity, sequence-specific DNA binding. The top ten of the KEGG pathway are pathways in cancer, proteoglycans in cancer, HIF-1 signaling pathway, bladder cancer, acute myeloid leukemia, pancreatic cancer, PI3K-Akt signaling pathway, ErbB signaling pathway, prostate cancer, choline metabolism in cancer. Pathways closely related to endocrine aging were extracted from the KEGG pathway, including pancreatic cancer, oxytocin signaling pathway, ErbB signaling pathway, HIF-1 signaling pathway, FoxO signaling pathway, prolactin signaling pathway, MAPK signaling pathway, thyroid hormone signaling pathway, insulin resistance, endocrine resistance, AMPK signaling pathway.

## 3. Changes in Structure and Function in Endocrine Glands during Aging

**Figure 4:**
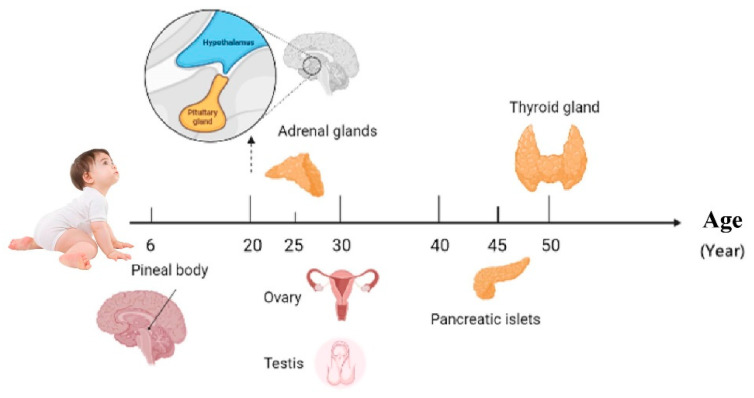
Endocrine glands aging sequence.

**Figure 5:**
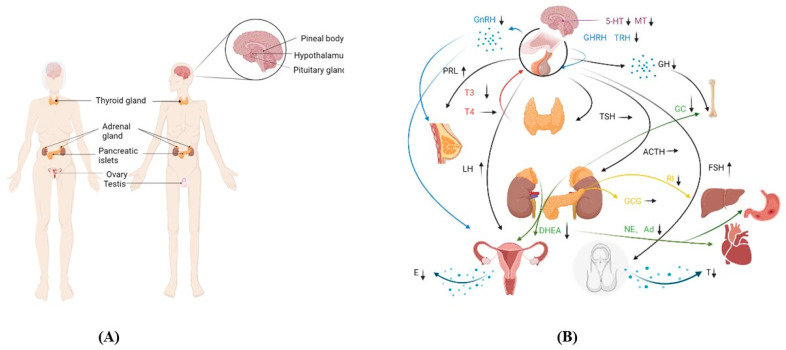
Changes in the function of endocrine glands. (**A**) Endocrine glands in the body. (**B**) Changes in hormone secretion and the action of endocrine organs. The same color curve represents the same endocrine gland, and the arrow is typically used for a particular organ.Abbreviations: 5-hydroxytryptamine (5-HT), melatonin (MT), gonadotropin-releasing hormone (GnRH), thyrotropin-releasing hormone (TRH), prolactin (PRL), growth hormone (GH), thyroid-stimulating hormone (TSH), adrenocorticotropic hormone (ACTH), follicle-stimulating hormone (FSH), triiodothyronine (T3), tetraiodothyronine (T4), dehydroepiandrosterone (DHEA), glucocorticoids (GC), noradrenalin (NE), adrenalin (Ad), insulin (INS), glucagon (GCG), estrogen (E), and testosterone (T).

The most significant sign of aging is the aging of the endocrine glands [4]; the timing sequence of aging is different (Figure 4) [5,6,7,8,9,10,11,12]. With the decline of endocrine glands, the levels of endocrine hormones are affected by each other (Figure 5). This chapter gives a general review of the structural and functional changes that occur when endocrine glands age, drawing a connection between aging and disorders that are connected to the decline of the endocrine glands (Table 1).

### 3.1. Central Endocrine Glands

#### 3.1.1. Hypothalamus

There are a group of special neuroendocrine cells in the hypothalamus that play an important role in regulating growth, development, metabolism, reproduction, and the homeostasis of the internal environment [13]. The suprachiasmatic nucleus (SCN) is located in the hypothalamus. The hypothalamus operates according to the photoperiod in order to stimulate internal functioning [14]. With age, abnormalities in the circadian rhythm occur, which in turn is accompanied by a failure of the hypothalamus in the first place [15]. Goyal and his colleagues revealed that the brain declined around the age of 20 [5]. The hypothalamus controls most of the brain’s operations. The hypothalamus is thus referred to as the “aging clock”.

**Figure 6:**
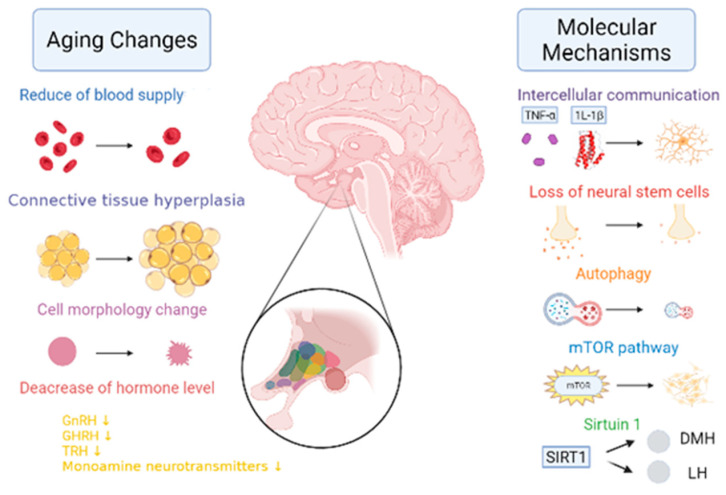
The changes and molecular mechanisms of hypothalamic aging. With aging, the hypothalamus undergoes the following changes in aging: a significant reduction in blood supply [16]; the proliferation of connective tissue [17]; a change in cell morphology [18]; a reduction in the gonadotropin-releasing hormone (GnRH) [20], the growth hormone releasing hormone (GHRH) [20], the thyrotropin-releasing hormone (TRH) [20], and in the monoamine neurotransmitter levels [23]. Molecular mechanisms of aging include the following: a blocked TNF-α and IL-1β signaling; the loss of neural stem cells [24]; reduced autophagy [25]; enhanced mTOR activity [26]; and reduced SIRT1 expression in SCN, thereby resulting in reduced dorsal medial hypothalamic (DMH) and lateral hypothalamic (LH) activity [27,28].

When the hypothalamus begins to age, the blood supply is significantly reduced, the connective tissue proliferates, and the cell morphology is irregular [16,17,18] (Figure 6). Its mechanism is found in the aging hypothalamus, which reduces ATG7 and LC3-II levels as well as the autophagic flow rates. Furthermore, p62 significantly accumulates in the POMC neurons, resulting in increased levels of POMC precursor protein and decreased α-MSH in the hypothalamus of aged mice [19]. In addition, Aβ activates NF-κB by selectively inducing the nuclear translocation of the p65 and p50 subunits, thereby repressing the gonadotropin-releasing hormone Ⅰ (GnRHⅠ) gene [20], thus resulting in a decreased GnRH. Due to positive and negative feedback regulation, growth hormone-releasing hormones (GHRHs) and thyroid-stimulating hormone-releasing hormones (TRHs) both decrease. According to the relevant literature research, by giving the NAL-GLU GnRH antagonist to healthy menopausal women between the ages of 48–57 and 70–77 years, the results demonstrated that the levels of GnRH in elderly women aged 70–77 years, were approximately 42% lower than those aged 48–57 years old [21]. The GHRH levels in 80 year olds were 70% lower than those found in 20 year olds [22]. The regulation of these hormone levels was inseparable from the secretion of monoamine transmitters by the hypothalamic monoaminergic neurons. However, with respect to 60-year-old women, when compared to the ones who were 20, the dopamine levels were decreased by 41%, serotonin by 43%, and norepinephrine by 30% [23]. 

Aging is accompanied by an abnormal energy regulation in the hypothalamus and glial cells. They are important players in the context of energy balance metabolism and are closely related to many metabolic diseases. In particular, cell populations within the arcuate nucleus of the hypothalamus sense the nutritional state of the organism and integrate signals from the peripheral hormones that are involved in glucose metabolism and energy expenditure [25]. Metabolic diseases are exacerbated by inflammatory activation under metabolic stress. When a glial cell is activated, it releases proinflammatory molecules, including interleukin-1β (IL-1β), interleukin-6 (IL-6), and the tumor necrosis factor-α (TNF-α) [24]. This significantly affects signaling, which leads to neuroinflammation. Hypothalamic inflammation alters insulin sensitivity in the peripheral tissues, increases the food intake involved in the development of obesity, and alters blood pressure regulation [29]. However, increased mTOR signaling results in increased POMC neuron cell sizes and reduced PVN neural projections. The injection of Rapamycin, a mTOR inhibitor, caused the stimulation and neuroprojection of POMC neurons and reduced food intake and weight [26]. The results illustrate the mTOR signaling pathway as one of the mechanisms regulating hypothalamic aging. In addition, hypothalamic inflammation is associated with central leptin resistance [30]. Thus, the development of the metabolic syndrome may be significantly influenced by hypothalamic inflammation. It is worth mentioning that the interaction between SIRT1 and the hypothalamic clock genes (i.e., *rBmal1, rPer1, rPer2, rCry1, rCry2*, and *rRev-erbα*) decreases with age, which would lead to reduced dorsal medial hypothalamic (DMH) and lateral hypothalamic (LH) activity [27,28].

#### 3.1.2. Pituitary

**Figure 7:**
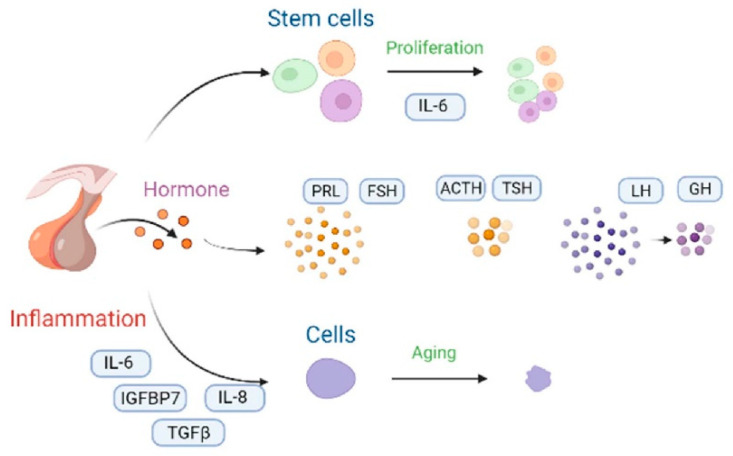
The changes and molecular mechanisms of the pituitary aging hormone. Proliferation of the pituitary stem cells can be enhanced by IL-6 supplementation, and they can also achieve anti-aging purposes [28]. When the levels of the pituitary aging hormones changed, the levels of prolactin (PRL) and the follicle-stimulating hormone (FSH) increased [32], whereas the adrenocorticotropic hormone (ACTH) and the thyroid-stimulating hormone (TSH) did not change significantly [34]. Moreover, the luteinizing hormone (LH) and the growth hormone (GH) levels decreased [31]. The inflammatory factors, including IL-6, IGHBP7, IL-8, and TGFβ activated the cellular senescence inflammatory network [36,37,38]. The black curved arrow represents the transformation method.

The pituitary gland secretes five main hormones, namely the growth hormone (GH), thyroid-stimulating hormone (TSH), and gonadotropins—including the luteinizing hormone (LH) and the follicle-stimulating hormone (FSH)—as well as the adrenocorticotropic hormone (ACTH) and prolactin (PRL) (Figure 7). GH dropped by 50% in the 60-year-old women [31]. Before menopause, elderly women had an increase in their FSH secretion by 76% compared with 30-year-old middle-aged women [32]. Furthermore, the FSH secretion of 65-year-old women increased by 50% after menopause [33]. The release of ACTH and TSH hardly changes with aging [34]. In fact, for the 20-year-old women, the pituitary stem cells show degenerative proliferative activation and up-regulated IL-6 injury [6]. Thus, pituitary stem cell capacity is activated. However, with aging, the inflammatory properties of senile glands increase, and the up-regulation ability of IL-6 damage is weakened, such that only organoids can be relied on to restore the activation ability of pituitary stem cells [35]. IGFBP7, TGFβ, IL-8, and IL-6 induce pituitary senescence due to an up-regulation of DNA damage as well as an activation of the p53 pathway [36,37,38]. Cells undergoing oncogene-induced senescence communicate with an inflammatory cytokine-producing environment. In addition, the transcription factor, C/EBPβ, cooperates with IL-6 in order to aggravate the inflammatory response [39]. Ultimately, the aging pituitary displays a notable reduction in mass, a reduction in blood supply, and an increase in connective tissue [40]. Eosinophils and basophils decrease due to a decrease in the number of effective divisions of nerve cells, and the pituitary gland displays more pronounced diffuse fibrosis and increases iron accumulation [41].

Severe aging pituitary glands can lead to hypopituitarism. The primary causes of this disease are pituitary cell destruction and anterior pituitary cell compression by larger adenomas, thereby resulting in gonadotropin, GH, TSH, and ACTH deficiency [42]. In addition, cardiovascular diseases and bone loss are inseparable causes of the aging of the pituitary gland. The women over 40 ovulated less frequently, while serum estradiol levels dropped, FSH levels increased, and LH levels stayed unchanged [43]. It is the changes in serum hormone concentrations that lead to an increased risk of cardiovascular disease and vasodilatory instability. Before menopause, the prevalence was lower in women than in men. However, after menopause, the prevalence was much higher in women than in men. Meanwhile, bone loss was exacerbated by reduced serum levels of estrogen due to the hypogonadism of the pituitary–gonadal axis. During perimenopause, women lost around 5–15% of their bone mass [44].

#### 3.1.3. Pineal Body

The pineal gland regulates the neuroendocrine and reproductive systems. Melatonin (MT), which is secreted by the pineal gland, aids with the regulation of the circadian rhythm and inhibits the secretion of gonadotropin. The pineal gland begins to calcify at birth, and melatonin synthesis starts to decline at age six [7]. The aging pineal gland is characterized by vascular narrowing, hardening, weight loss, decreased cell number, and lower melatonin levels. Reduced MT secretion is due to a reduced density of beta-adrenergic receptors in the pineal gland as well as the down-regulation of the *AANAT/SNAT* gene expression or phosphorylation [45]. Another reason is the increased consumption of MT due to metabolic reasons. For example, senescent cells produce more ROS than younger cells, and MT is used as a regenerative antioxidant to neutralize excessive ROS production in older organisms [46]. MT is a direct free radical scavenger with more powerful antioxidant potential than traditional antioxidants, such as vitamins C and E, as well as mannitol and glutathione [47]. It can quickly remove damaging free radicals in the aging body, and then play a role in delaying aging.

The organism can suffer significant harm when MT secretion is diminished. MT regulates *LC3-Ⅱ, Beclin-1, P62, CDK5, and GSK3*. In addition, it activates the mTOR pathway, inhibits α-nucleoprotein, reduces Tau phosphorylation, and clears Aβ accumulation, reducing Aβ toxicity in order to regulate autophagy, and thereby protecting the nervous system [48]. However, when MT secretion begins to decrease, the ability to regulate autophagy decreases, thus increasing the risk of neurodegenerative diseases. In addition, the reduction in endogenous MT will lead to decreased neuronal resistance to oxidative stresses or brain inflammations [49]. Impaired amounts of 5-hydroxytryptamine, its derivatives, and polypeptide hormones are produced as a result of lower neuronal resistance to oxidative stress and brain inflammation, which, in turn, reduces the body regulatory activities and, as a result, the delayed reaction in the aged. Additionally, MT is crucial for postponing ovarian aging. Furthermore, MT administration inhibits follicular growth and atresia as well as various pathways that are involved in follicle formation, including *PI3KAKT* [50]. MT stimulates angiogenesis and increases VEGF and VEGF-R1 expression in the ovaries [51]. In summary, it is evident that decreased MT secretion will hasten ovarian aging and postpone conception.

### 3.2. Peripheral Endocrine Glands

#### 3.2.1. Thyroid

The thyroid gland begins to age more quickly around age 50 [8]. It begins to undergo fibrosis and atrophy, resulting in a significant reduction in its size and weight by 40% to 60% [8]. The number, size, and secreted particles of the follicles are reduced, and the glial staining in the follicles is abnormal. The overall level of thyroid hormones decrease. For women who are 60 years old, T3 decreases by 25% to 40%. However, there was no significant change in the T4. That may be due to the TH clearance rate and the decreased secretion [52]. However, T3 decrease may be due to a decreased basal metabolic rate, thereby resulting in a decrease in the peripheral conversion rate of T4 to T3. Up to 6% of subjects over 65-years-old suffer from significant hypothyroidism, and the prevalence of women was higher than that of men [53]. 

An aging thyroid gland and hormonal abnormalities will cause significant adverse effects on the body. For example, thyroid hormones have a role in regulating heartbeat, pulse, blood circulation, heart contraction, and the rate of oxygen consumption [54]. When thyroid hormone levels are abnormal, primarily T3, there will be adverse effects on the heart and cardiovascular system. Patients may initially experience low blood pressure with thyroid hormone deficiency. After a while, blood pressure rises, resulting in atherosclerosis as well as heart attack and stroke. The rationale boils down to the fact that thyroid hormones are closely linked to genes that regulate the contractile function of the heart, including sarcoplasmic reticulum (SR) Ca^2+^ ATPase (SERCA2), phospholamban (PLB), and the myosin heavy chains (MHC), α and β [54]. Some of these genes are positively regulated (SERCA2, α-MHC) whereas others are negatively regulated (PLB, β-MHC) by T3 [54,55]. In addition, the effects of thyroid hormones on cardiac myocyte-specific gene transcription were mediated by thyroid hormone receptors (TRs) [56]. Generally, TRs activate the transcription of positively regulated genes in the presence of T3 by recruiting coactivator complexes and repressing transcription in the absence of ligands by recruiting corepressor complexes [56,57]. Therefore, low levels of thyroid hormones will induce changes in blood pressure.

#### 3.2.2. Adrenal Glands

After age 20, fibrosis, weight loss, cortical nodules, cortical and medullary cells, and connective tissue hyperplasia are evident in adrenal glands [9]. The reticular zone (ZR) thickness reduces, but the total thickness of the adrenal cortex does not alter significantly [58]. The cortical globular zone shrinks, the levels of glucocorticoids decrease, renin activity decreases, and the production of renin-angiotensin II decreases, thereby leading to a decrease in aldosterone content. Catecholamine levels decrease due to lower norepinephrine and epinephrine levels. In a study of aging mice, the ratio of dopamine to norepinephrine was lower in 24-month-old mice than those found in 12-month-old mice. In addition, the ratio of norepinephrine to epinephrine was significantly lower at 24 months of age than at 6 months of age. Adrenal levels were 17% and 37% lower at 12 and 24 months of age, respectively, than at 6 months of age [59]. In addition, dehydroepiandrosterone (DHEA) and dehydroepiandrosterone sulfate (DHEAS) reached secreted peaks in 20-month-old mice and then began to plummet in 25-month-old mice [59]. When the age of 80 years was reached, the content of DHEA was reduced from 10% to 20% [60]. However, Lasley and his colleagues reported that the overall level of DHEAS first showed an increase and then a decrease with age. They found that in around 85% of women, adrenal androgen production rose during the menopausal transition, which starts in early perimenopause and continues into the early post-menopause [61]. Elevated DHEAS is attributed to adrenals, not ovaries [62]; however, the decrease in DHEAS levels after menopause is influenced by ovarian regulation [63].

DHEA and DHEAS are both major steroids that are secreted by the adrenal glands and play a role in musculoskeletal integrity, sexual function, emotional aspects, cognitive performance, and cardiovascular health. Numerous investigations in the context of the musculoskeletal system demonstrate a beneficial correlation between blood DHEA levels and musculoskeletal integrity [64,65,66]. Low DHEAS levels will cause fatigue, back pain, and weakness in the body. In terms of sexual function, women with low serum DHEAS levels have a low sexual response. Receiving oral DHEA replacement therapy may enhance sexual interest [67]. In terms of mood, DHEA modulates the release of dopamine, glutamate, and c-aminobutyric acid, as well as inducing increased 5-hydroxytryptamine (5-HT) neuronal activity [68]. It can be concluded that low DHEA levels can lead to depressed moods and severe depression. In terms of cognitive performance, low DHEA/DHEAS levels are thought to contribute to the onset of Parkinson’s disease, which is closely associated with DHEA-induced production of the pro-inflammatory factors, TNF-α and IL-6 [69]. In cardiovascular health, low DHEA/DHEAS levels may induce the development of atherosclerosis and cardiovascular disease [70].

It is worth noting that glucocorticoids are also an important hormone secreted by the adrenal glands. Glucocorticoids act as a bridge between the suprachiasmatic master clock and almost all peripheral clocks. When the adrenal cortex function is decreased, glucocorticoid level is decreased, which renders it easy to then cause the circadian rhythm disorder. For example, in a study of glucocorticoid-induced clock gene expressions in mice, it was demonstrated that glucocorticoids could regulate the circadian rhythm gene, Bmal1 [71]. Interestingly, glucocorticoid-induced phase shifting did not occur in PER1 knockout models. This suggests that PER plays a major role in mediating the effects of glucocorticoids on other circadian components of the clock mechanism, such as Bmal1, Rev-Erb, and Clock [72]. It has been reported that exogenous glucocorticoid injection was used to treat the related diseases caused by adrenal hypofunction, including circadian rhythm disorders. It has been shown to increase the incidence and mortality of cardiovascular disease and malignancy [73,74]. However, most guidelines recommend that less than 30 mg of glucocorticoids are used to treat patients with hypoadrenal function [75]. These two conclusions are contrary to each other and are presumed to be closely related to the time and dose of the administration, which requires further clinical data to prove.

### 3.3. Endocrine Tissue

#### 3.3.1. Ovary

**Figure 8:**
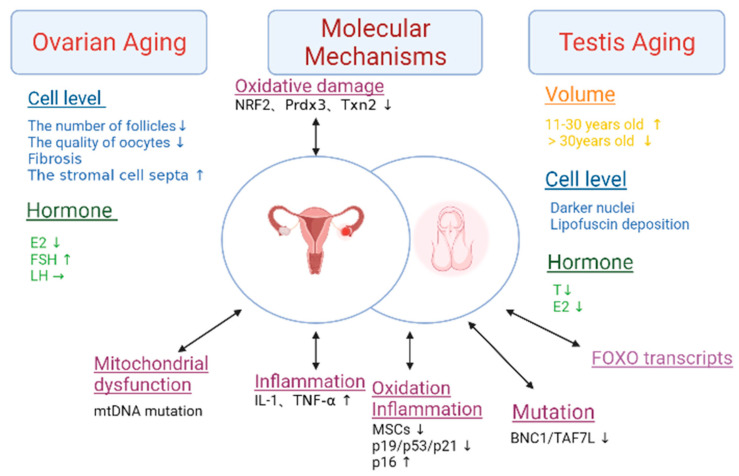
The characteristics and molecular mechanisms of gonads aging. This figure summarizes the changes in volume, cellular levels, and hormone levels in the aging ovary and testis. The mechanisms of aging in the ovary include oxidative damage, mitochondrial dysfunction, and inflammation. The mechanisms of aging in the testis include oxidation, inflammation, mutation and FOXO transcripts. Abbreviations: estradiol (E2), the follicle-stimulating hormone (FSH), the luteinizing hormone (LH), testosterone (T), interleukin-1 (IL-1), tumor necrosis factor-alpha (TNF-a), and mesenchymal stem cells (MSCs). The double arrows represent the two-way mode of action.

After the age of 30, ovarian function declines significantly, resulting in a decrease in follicle number and oocyte quality [10]. This results in the down-regulation of the *Prdx3* and *Txn2* genes, which ultimately leads to a decline in female fertility [10] (Figure 8). When women reach 45, fibrosis begins to occur, the uterus and vagina atrophy, and they also lose weight and shrink into smaller pieces of connective tissue. In addition, the stromal cell septa expand. In addition, the medulla and cortex decrease in volume. The swellings on the surface of the ovaries disappear and are flat, with no follicles on the surface, and they also occasionally lose vesicles. The cortex has only a few atresia or cystic follicles, which are rich in connective tissue. Calcinosis or old hemorrhage can be seen, and the blood vessels show signs of sclerosis. Estrogen and progesterone are rapidly reduced. The menstrual cycle gradually develops from disorder to menopause. Under normal circumstances, women enter the menopausal stage around the age of 60. At the age of 40, the frequency of ovulation decreases, and ovarian function ceases within 15 years [76]. During this period, the function of the follicles is extremely poor, and the ovaries no longer secrete estrogen and estradiol (E2). These hormones are only supplied by androstenedione and interstitial ovarian cells in the adrenal cortex, but the total amount is significantly reduced. The concentration of FSH is higher than that of young women, and there was almost no change in LH. 

Estrogen plays an essential role in maintaining bone health, ovarian function, and youthful skin condition. Low levels of estrogen will cause osteoporosis. The reason for this is that estrogen induces Sema3A expression in osteoblasts. This process reduces osteoclasts while increasing osteoblasts and prolonging the survival of osteoclasts, thus resisting bone aging [77,78]. Estrogen is the most important component for the function of the ovaries. When estrogen levels decrease, it will inhibit follicle development [79]. It indirectly impacts ovarian function by preventing gonadotropin release through the positive and negative feedback control of the hypothalamus [80]. In addition, lower estrogen levels accelerate the aging of the skin. This is because estrogen enhances the expression of TGF-β to promote the production of subcutaneous VEGF. It can upregulate the expression of TIMPs and down-regulate the expression of MMPs, which reduces the degradation of collagen. It also inhibits FLT3 kinase activity and reduces Ras/mitogen-activated protein kinase/extracellular signal-regulated kinase and Akt/p70 ribosomal S6 kinase pathways [81]. This inhibits the activity of AP-1 and reduces the gene transcription of MMP-1, which ultimately delays skin aging [82]. In conclusion, estrogen plays a vital role in the organism. Currently, estrogen replacement therapy is widely used, but clinical data remains inadequate. Certain studies suggest that this method may increase breast and endometrial cancer risk [83,84]. Therefore, further clinical trials are needed to prove this.

#### 3.3.2. Testis

The average testis volume increases between men who are 11–30 years old and only decreases in those who are 30–60 years old, specifically around 2.8%. After reaching 60 years of age, the average testis volume decreases at a rate of 0.42 cm^3^/y [85]. After 30 years, the testis will see the thickening of the seminiferous tubules and basement membrane, a decrease in seminiferous epithelial cells, and the narrowing and hardening of the lumen [11]. The number of testicular stromal cells decreases and become steroidogenically hypofunctional [86]. Compared with normal cells, the testicular stromal cells of elderly men have darker nuclei and more irregular shapes, and may also contain an underdeveloped endoplasmic reticulum, with many lipofuscin particles attached to the surface [87]. Leydig cell counts are maintained, but these cells may have less testosterone synthesis capacity, which would lower blood testosterone levels. Additionally, there is a reduction in the amount of androgen secreted and a weakening of the testicular response to gonadotropin stimulation. Men begin to experience a decrease in serum total testosterone and free testosterone levels from the age of 50–59. Compared with those who are 20-years-old, when they men are 80, the total testosterone level in the serum drops by 35% and the free testosterone level drops by 50% [88]. A total of 810 males between the ages of 24 and 90 had their plasma total testosterone, bioavailable estradiol, and estradiol levels examined. It was discovered that the total estradiol level was reduced by 0.03 pg/mL and the bioavailable estradiol level decreased by 0.12 pg/mL [89]. The metabolic rate of the testicles, similarly, exhibited a negative tendency, declining by about 10.9% by age 60 when compared to age 40 and by about 10% by age 70 when compared to age 60 [85]. That may be related to the increase in gonadotropin levels and the decrease in free testosterone in serum. The LH reduced and weakened following the stimulation of the testes, which, in turn, led to a decrease in total serum testosterone. 

The development of atherosclerosis, coronary artery disease, and cardiovascular disease is strongly associated with low testosterone levels. Patients with hypotestosteronemia have considerably lower levels of the anti-inflammatory cytokine IL-10 and significantly higher levels of the pro-inflammatory cytokines, TNF and IL-6 [90]. This could be proof that atherosclerosis and inflammation are brought on by low testosterone levels. Studies have shown that testosterone supplementation therapy can improve blood glucose and insulin resistance [91]. Therefore, coronary artery diseases, such as diabetes, are associated with low testosterone levels. Additionally, testosterone levels are adversely connected with atherogenic low-density lipoprotein (LDL) cholesterol and triglycerides as well as favorably correlated with cardioprotective high-density lipoprotein (HDL) cholesterol [92,93]. In a review by Farid and his colleagues, it was noted that testosterone treatment had beneficial effects on body composition, lipid levels, blood glucose control, and blood pressure. In addition, it may reduce the risk of cardiovascular disease [94]. This is explained by the fact that testosterone inhibits VSMC aging and collagen formation by acting on the Akt/FoxO1a- and growth arrest-specific protein 6 (Gas6)/Axl pathways [95]. Testosterone treatment restores phosphorylation of the Akt and FoxO1a pathways. Furthermore, it induces serum testosterone and Gas6 levels, and ameliorates cellular senescence and vascular remodeling [96].

#### 3.3.3. Pancreatic Islets

Pancreatic islets mainly play the role of secreting insulin and glucagon to regulate the body’s blood sugar balance. With aging, the pancreatic islets shrink and the islet cells degenerate. The secretion of glucagon and the responsiveness of stimulation hardly change. The number of β-cells decreases and atrophy occurs. In addition, the process also includes lipofuscin deposits and insulin content decreases. There is another argument that insulin content is not affected by age and that endogenous insulin sensitivity is reduced by 40% [97]. 

Type 2 diabetes (T2D) is the most typical age-related disease that is caused by the aging of pancreatic islets. Insulin resistance and β-cells dysfunction are two major causes of T2D pathology. The pathological features of pancreatic islets in insulin-dependent T2D mellitus are characterized by acinar cell atrophy, stromal fibrosis, atherosclerosis, and obstructive ductal lesions with epithelial hyperplasia or dysplasia [98]. In addition, T2D patients showed significant histological features of inflammation in the islets, including the infiltration of immune cells [99], amyloid deposits [100], cell death, and fibrosis [101]. The reason may be the decrease in pancreatic islets’ replication function, the decrease in the insulin secretion ability, the decrease in the self-repair ability after damage, and the increase in cell apoptosis, which lead to the obvious decline of the ability of cells to withstand metabolic pressure or even to withstand the occurrence of diabetes [102]. In addition, observations in certain rodent models and humans have clearly shown that pancreatic islets’ macrophages induce β-cell dysfunction by secreting IL-1 [103,104]. Studies have also revealed that certain T2D patients develop islet autoimmunity during the course of the disease, which leads to decreased beta cell function [105]. Therefore, mediating islet inflammatory cells will help improve type 2 diabetes. The risk of diabetes increases rapidly after the age of 45 years [12]. Based on this, it has been hypothesized that pancreatic islets aging is accelerated after 45 years of age.

## 4. Conclusions

The endocrine theory is a hot topic of research into the mechanisms of aging. Many anti-aging and age-related disease medications act to regulate endocrine hormone levels. Therefore, the endocrine glands have been defined as the key factors influencing the aging of the body. This leads to age-related diseases. The problem as to how to slow down aging is then the most urgent challenge to be faced. 

In order to further promote aging research and to develop safer and more effective anti-aging medication, this article outlines the timing sequence and mechanisms of aging in endocrine glands. Particular emphasis is placed on the following. (1.) There were 44 genes related to endocrine aging. The most dominant KEGG pathway is the MAPK signaling pathway. (2.) The first endocrine organ to undergo aging is the pineal gland, at around 6 years old. This is followed, in order, by the hypothalamus, pituitary gland, adrenal glands, gonads (ovary and testis), pancreatic islets, and the thyroid gland. (3.) When endocrine glands fail, they generally show structural and functional changes, such as loss of mass, abnormal hormone secretion, and cellular deformation. (4.) The aging mechanism of endocrine glands is closely related to oxidative stress, cellular levels, gene levels, and hormone levels. 

It is hoped that this review will provide the reader with a framework for further research into endocrine glandular aging.

## Figures and Tables

**Table 1:** Changes in endocrine glands during aging. (↓ represents decrease; ↑ represents increase; → represents unchange.).

Endocrine Glands	Function	Structural Change	Changes in Secretory Hormones	Influence on Organism	References
Hypothalamus	Regulating the nervous system, water metabolism, salt metabolism, temperature regulation, food intake, sleep, reproduction, visceral activities, and emotions	Reduced mass, reduced blood supply, hyperplasia of connective tissue and changes in cell morphology	GnRH ↓ 42% GHRH ↓ 70% TRH ↓ 10%; Monoamine neurotransmitters ↓	Insomnia, mental retardation, depression, obesity, diabetes, hypertension, and other diseases	[5,13,14,15,16,17,18,19,20,21,22,23,24,25,26,27,28,29,30]
Pituitary	Regulating growth, development, reproduction, metabolism, stress, and aging	Decreased mass, decreased blood supply, and increased connective tissue	GH ↓ 50%. Before menopause, FSH ↑ 76%. After menopause, FSH ↑ 50%. PRL ↑ 128%. ACTH and TSH → LH first ↑ and then ↓	Reduction in skeletal development, diabetes insipidus, obesity, and microadenoma	[6,31,32,33,34,35,36,37,38,39,40,41,42,43,44]
Pineal body	Regulating the secretion of the nerve and reproductive system	Vascular narrowing, hardening, weight loss, reduced cell number, and other phenomena	5-HT and its derivatives ↓ Polypeptide hormones ↓ Melatonin →	Decreased sleep quality and slow responses	[7,45,46,47,48,49,50,51]
Thyroid	Promoting body organ development and central nervous system maturation	Thyroid fibrosis and atrophy ↓ 40–60%. The number and size of follicles and the secreted particles are reduced. The glial staining in the follicles is abnormal	T3 ↓ 25~40% T4 →	Arrhythmia and myocardial infarction, hyperthyroidism, and hypothyroidis	[8,52,53,54,54,55,56,57]
Adrenal glands	Regulating body metabolism	Fibrosis, weight loss, cortical nodules, cortical and medullary cell loss, and connective tissue hyperplasia	DHEA ↓ 20% Cortisol, adrenal, glucocorticoids, rostenedione, and aldosterone ↓	Osteoporosis, diabetes and tumors	[9,58,59,60,61,62,63,64,65,66,67,68,69,70,71,72,73,74,75]
Ovary	Ovulation and secretion of female hormones	Volume atrophy, weight loss, hypofunction, and vascular sclerosis	E2 ↓ Androgen polypeptide hormones ↓	Menopause syndrome and postmenopausal osteoporosis	[10,76,77,78,79,80,81,82,83,84]
Testis	Producing sperm and secreting androgen	Thickening of lamina propria and the basement membrane of seminiferous tubules. A reduction in spermatogenic epithelial cells, as well as the narrowing and hardening of the lumens	T ↓ 35% E2 ↓ 0.03pg/mL Bioavailable E2 ↓ 0.12 pg/mL	Decreased muscle strength, osteoporosis, decreased libido, cardiovascular disease	[11,85,86,87,88,89,90,91,92,93,94,95,96]
Pancreatic islets	Secreting insulin and glucagon, as well as synthetic metabolism and regulating blood glucose stability	Pancreatic atrophy, reduced number of islet cells and degeneration	Glucagon → Lipofuscin deposition Insulin↓or →	Diabetes and stress hyperglycemia	[12,97,98,99,100,101,102,103,104,105]

## Data Availability

Not applicable.
